# The tammar wallaby major histocompatibility complex shows evidence of past genomic instability

**DOI:** 10.1186/1471-2164-12-421

**Published:** 2011-08-19

**Authors:** Hannah V Siddle, Janine E Deakin, Penny Coggill, Laurens G Whilming, Jennifer Harrow, Jim Kaufman, Stephan Beck, Katherine Belov

**Affiliations:** 1 Faculty of Veterinary Science, University of Sydney, NSW 2006, AUSTRALIA; 2 ARC Centre of Excellence for Kangaroo Genomics, Research School of Biological Sciences, Australian National University, Canberra, ACT 0200, Australia; 3 Wellcome Trust Sanger Institute, Wellcome Trust Genome Campus, Hinxton Hall, Hinxton, Cambridgeshire, CB10 1SA, UK; 4 University of Cambridge, Department of Pathology, Cambridge CB2 1QP, UK; 5 UCL Cancer Institute, University College London, London WC1E 6BT, UK

## Abstract

**Background:**

The major histocompatibility complex (MHC) is a group of genes with a variety of roles in the innate and adaptive immune responses. MHC genes form a genetically linked cluster in eutherian mammals, an organization that is thought to confer functional and evolutionary advantages to the immune system. The tammar wallaby *(Macropus eugenii*), an Australian marsupial, provides a unique model for understanding MHC gene evolution, as many of its antigen presenting genes are not linked to the MHC, but are scattered around the genome.

**Results:**

Here we describe the 'core' tammar wallaby MHC region on chromosome 2q by ordering and sequencing 33 BAC clones, covering over 4.5 MB and containing 129 genes. When compared to the MHC region of the South American opossum, eutherian mammals and non-mammals, the wallaby MHC has a novel gene organization. The wallaby has undergone an expansion of MHC class II genes, which are separated into two clusters by the class III genes. The antigen processing genes have undergone duplication, resulting in two copies of TAP1 and three copies of TAP2. Notably, Kangaroo Endogenous Retroviral Elements are present within the region and may have contributed to the genomic instability.

**Conclusions:**

The wallaby MHC has been extensively remodeled since the American and Australian marsupials last shared a common ancestor. The instability is characterized by the movement of antigen presenting genes away from the core MHC, most likely via the presence and activity of retroviral elements. We propose that the movement of class II genes away from the ancestral class II region has allowed this gene family to expand and diversify in the wallaby. The duplication of TAP genes in the wallaby MHC makes this species a unique model organism for studying the relationship between MHC gene organization and function.

## Background

The major histocompatibility complex (MHC) is a group of immune genes critical for immune response to pathogens, immunoregulation, anti-tumour responses and inflammation. Disease resistance and susceptibility associations have been identified between MHC genes and autoimmune diseases [1], infectious diseases [2] and parasite load [3]. Although MHC genes have been found in all jawed vertebrates, the region is dynamic and MHC genes have been reorganized throughout vertebrate evolution as species evolve and adapt to new pathogenic and environmental pressures [4,5].

The MHC of eutherian mammals is a large cluster of linked genes, broadly divided into three regions, class I, class II and class III. These regions are named for the primary type of MHC gene found within them. The class I and class II MHC genes encode molecules responsible for antigen presentation. The class I region contains class I genes, which present endogenous peptides to CD8+ T cells, and also contains a collection of well conserved genes with varying functions known as the framework genes, including the members of the TRIM family of genes, FLOT1, TUBB and NRM [6]. The class II region contains class II genes, which present exogenous peptides to CD4+ T cells. This region also contains the antigen processing genes, including TAP (transporter associated with antigen processing), PSMB/LMP (Large mutli-functional proteasome) genes and non-classical class II genes belonging to the DM and DO gene families. TAP molecules are encoded by two genes, TAP1 and TAP2, which are transmembrane proteins that form a heterodimer within endoplasmic reticulum (ER) membrane, where they transport peptides from the cytosol to the ER to be coupled to class I molecules [7]. The DM and DO molecules stabilize peptide binding to class II molecules. The class III genes are so called due to their position between the class I and class II regions. These genes do not have a homogenous function, but many have roles related to the innate immune response (for example tumour necrosis factor (TNF) and lymphotoxin α and β, LTA and LTB) [8].

The human MHC spans 3.6 Mb and includes 264 genes [9], with the MHC of most other eutherians spanning a similar genetic area and gene richness [5]. In eutherian mammals the three MHC regions are linked, with the class I and class II regions separated by the class III region. The organization of MHC genes is also generally conserved in eutherian mammals, but with some variations, including the presence of classical class I genes adjacent to the antigen processing genes in the rat [10] and the separation of the class II region from the remainder of the MHC in pigs [11]. Despite these variations, linkage of MHC genes is thought to provide functional advantages via co-evolution of genes, generation of diversity and co-ordination of expression and function [12].

Among non-mammals a diversity of MHC 'shapes and sizes' has been identified. For example, the MHC region of the chicken (the B locus) is considered to be 'minimal essential' spanning 92 Kb and containing only 19 genes [13]. In multiple lineages of teleost fish the class I and class II genes are not linked, and the class III genes are fragmented across multiple chromosomes [14]. In contrast, the MHC of the amphibian, *Xenopus *shows some similarity to the human MHC, with many class III genes assembled in a similar gene order to the human MHC [15]. Despite this diversity, a common feature of the non-mammalian MHC is that the class I genes are found adjacent to, or interspersed with, the antigen processing genes [16], which generally are found adjacent to classical and non-classical class II genes. This organization is thought to provide an advantage in that the antigen presenting and antigen processing genes can then co-evolve, with little recombination between them [17]. The tight linkage of the antigen processing and antigen presenting genes has been retained to varying degrees in extant mammals and non-mammals [5].

Characterizing the MHC of distant mammals will provide insights into how the MHC evolved in vertebrates. Marsupials and eutherians last shared a common ancestor approximately 148 million years ago, and since then their immune systems have been evolving independently under different pathogenic pressures, making marsupials ideal for comparative studies of the MHC region. We previously annotated the MHC of the grey short-tailed opossum (*Monodelphis domestica*) [18], the first marsupial to have its genome sequenced and found that the opossum class I genes were interspersed with the antigen processing genes and class II genes. This organization is similar to that of many non-mammalian species [15,18]. However, the opossum class III genes and framework genes that flank the eutherian class I genes are found in a similar order to those in eutherians. The opossum class II genes fall into four gene families, with DA, DB and DC gene families thought to be unique to marsupials [19] and the non-classical DM family shared with other mammalian and non-mammalian species [18]. There are 13 putative opossum class I genes. One of these genes (*Modo-UA*) likely has a classical function of antigen presentation as it is ubiquitously expressed and highly polymorphic. Two class I genes, which are closely related to *Modo-UA, Modo-UB *and *Modo-UC*, are found outside the MHC [18,20]. Whether these genes encode molecules with a classical role in antigen presentation remains unclear, but they are expressed with unknown levels of polymorphism [18]. Aside from *Modo-UA*, six other MHC linked class I genes are transcribed. All six genes appear to be non-classical, lacking polymorphism and with tissue specific expression, but their functional roles remain to be determined [21]. The opossum MHC has one TAP1 gene, two TAP2 (TAP2A and TAP2B) genes and a PSMB8 and PSMB9 gene, but it is not known which of these is expressed.

Comparison of the opossum MHC with that of another marsupial species is important as marsupials are an evolutionarily diverse group with orders in both South America and Australia. The tammar wallaby (*Macropus eugenii*) is an Australian macropod, which last shared a common ancestor with the opossum ~80 mya [22], a similar evolutionary distance as human and mouse. We recently showed that the organization of the tammar wallaby MHC is unique among vertebrates. Nine class I genes were found outside the MHC [23]. Seven of these appear to have a classical role in antigen presentation [24]. The non-classical MHC class I, classical MHC class II and class III genes have been mapped by FISH to chromosome 2q. Here we present a Bacterial Artificial Chromosome (BAC) contig and sequence of the tammar wallaby MHC. We show that the wallaby class I genes, antigen processing genes and class II genes have undergone extensive rearrangement when compared to the opossum and provide insights into the evolution of the mammalian MHC.

## Results

### MHC gene organization in the wallaby

**Figure 1:**
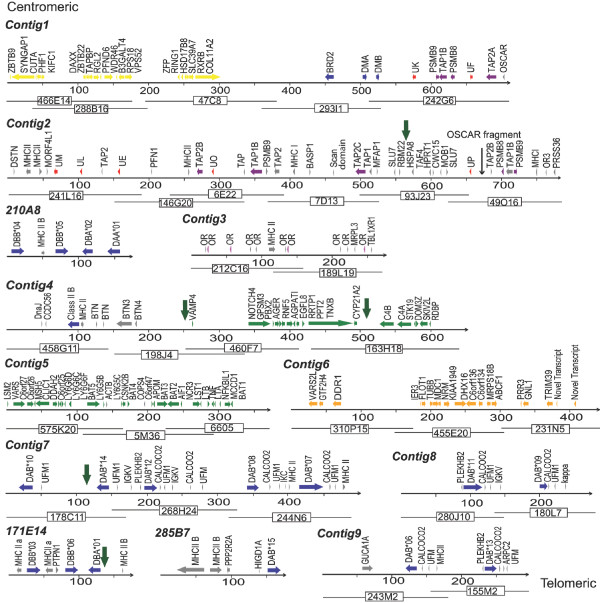
**Diagram of the organization of the wallaby MHC with gene annotation**. Colour code for genes; yellow - extended class II, blue - class II, red - class I, purple - antigen processing genes, pink - olfactory receptors, grey - pseudogenes. The overlapping BACs are indicated by black lines below the annotation. BACs not assembled into a contig are indicated by the BAC name. The KERV fragments are indicated by thick black arrows. An OSCAR fragment is indicated with an arrow.

**Figure 2:**
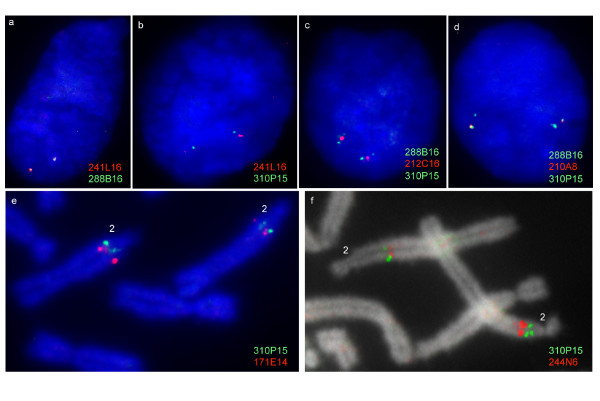
**Metaphase and interphase FISH showing the location of anchored BACs in order to order the contigs**. (a-d) FISH on tammar walaby interphase nuclei showing (a) co-localization of contig 2 (BAC_241L16 in red) and contig 1 (BAC_288B16 in green), b) the positions of contig 2 (BAC_241L16 in red) and contig 6 (BAC_310P15 in green) relative to one another, c) the position of BAC_212C16 relative to contig 1 (BAC_288B16) and contig 6 (BAC_310P15), BAC_212C16 is midway between the two contigs, d) the position of BAC_210A8 relative to contig 1 (BAC_288B16) and contig 6 (BAC_310P15), BAC_210A8 is closer to BAC_288B16 than BAC_310P15. e) Metaphase FISH showing that contig 4 (BAC_244N6 in red) is telomeric to contig 6 (BAC_310P15 in green), f) Metaphase FISH showing that BAC_171E14 in red is approximately 1 Mb telomeric to contig 6 (BAC_310P15 in green).

In total 4.7 Mb of tammar wallaby MHC sequence containing predictions for 129 putative functional genes has been generated from chromosome 2q. The BACs assemble into nine contigs plus three orphaned BACs (summarized in Table 1 and Figure 1). The contigs contain MHC class I, class II and class III genes, as well as genes from the extended class II region, antigen processing genes and framework genes. The three orphan BACs do not overlap with any contig, but contain additional class II genes and have been maped to chromosome 2q using FISH. The wallaby MHC genes have been ordered on chromosome 2q, using gene sequence and metaphase and interphase FISH (Figure 2).

**Table 1:** Summary of contigs across chromosome 2q

Contig/BAC	Size	No. of coding genes
1	711351	29

**2**	784023	11

**3**	276581	4

**4**	607028	20

**5**	370000	29

**6**	442620	19

**7**	494000	5

**8**	250000	2

**9**	282000	2

**210A8**	15600	4

**285B7**	170341	1

**171E14**	167000	3

**Total**	**4720944**	**129**

**Figure 3:**
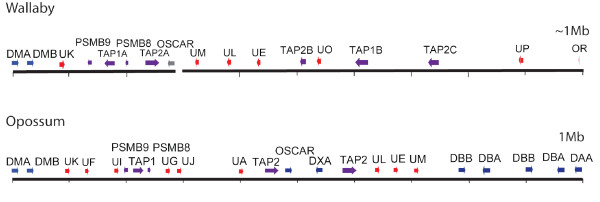
**Schematic comparison of the opossum MHC class I/II region and the putative class I/II region in the tammar wallaby**. Colour code for genes; blue-class II, red-class I, purple-antigen processing genes, grey-pseudogenes. Each 100 Kb is represented by a short black line.

Contig 1 covers a 711 Kb region and includes the extended class II region, class II DM α and β genes, a single class I gene, several antigen processing (TAP1 and TAP2) and proteasome genes (PSMB8 and PSMB9) (Figure 1). The gene content and order of the extended class II region from *Syngap1 *to *VPS52 *is almost identical between the opossum and the tammar wallaby and shares high similarity with the extended class II region of eutherian mammals. A key exception is the region around the class II DM genes, while there is ~350 Kb between the *DMB *and *OSCAR *genes in the opossum there is only 180 Kb in the tammar wallaby and as a result there are fewer genes within this region in the wallaby (Figure 3). In the opossum, this region contains five class I genes *Modo-UF, -UI, -UG, -UJ *(all non-classical class I genes) and *Modo-UA *(the single classical class I gene) that are not present within this region in the wallaby. In the wallaby, only a single non-classical class I gene (*Maeu-UK*) remains. The last gene in Contig 1 is an *OSCAR *(osteoclast-associated receptor) pseudogene. No evidence could be found for the first two exons of the OSCAR gene and homology (nucleotide and protein) to the human and mouse OSCAR genes terminates part way through the final exon. *OSCAR *is also found in the MHC of the opossum, adjacent to TAP2 and *Modo-UA*, but is found outside the MHC in eutherians.

Contig 2 covers a 784 kb region and contains class I and antigen processing genes. It does not overlap Contig 1 but based on interphase FISH results it lies adjacent (Figure 2). The region covered by Contig 2 represents the remnants of a class I/II region, with class I genes interspersed with antigen processing genes. The contig contains non-classical class I genes (*Maeu-UL, Maeu-UE*, *Maeu-UM *and *Maeu-UP*) as well as antigen processing genes *TAP1*, *TAP2*, *PSMB8 *and *PSMB9*. One putatively functional TAP1 and two putatively functional TAP2 genes were identified, as well as two TAP1 and four TAP2 pseudogenes (either gene fragments or with in-frame stop codons). Two *PSMB8 *and two *PSMB9 *genes and multiple PSMB pseudogenes were detected.

In the opossum a class II DBB gene (*Modo-DBB1*) is found 50 kb away from *Modo-UM*. In the wallaby a class II pseudogene that shares high similarity to expressed wallaby DBB genes [25] (Figure 3) is found 20 Kb away from *Maeu-UM *on Contig 2. We have a gap in our BAC contig in this region, but predict that the orphan BAC (210A8) containing DBB, DBA and DAA genes is in the region adjacent to Contig 2, based on FISH data (Figure 1 and 2) and the fact that this region in the wallaby appears to have once contained the class I/II region and the class II DM genes. The presence of an *OSCAR *fragment at one end of Contig 2 (BAC_49O16) suggests that this region was once part of Contig 1, but was rearranged, causing OSCAR to become a pseudogene in the wallaby.

Contig 3, a minicontig of ~300 kb, contains a class II DAB processed pseudogene and a cluster of olfactory receptor genes and has been mapped by FISH to the region between Contig 2 and Contig 4 (Figure 1 and 2). The processed pseudogene is intronless, including all exons (except the signal sequence), an in-frame stop codon and a putative polyA tail 700 bp downstream of the stop codon with a possible consensus sequence (AATTAAA) immediately upstream. Interphase FISH indicates that the signals for these contigs are indistinguishable and we estimate that the distance between Contigs 2 and 3 is less than 500 kb.

Contigs 4 and 5 contain 44 class III genes, class II genes belonging to the DC gene family and a cluster of butyrophilin (BTN) genes. The gene content and order of the 44 class III genes is nearly identical to that of the opossum. Comparison of Contigs 4 and 5 with the opossum class III region suggests that the contigs are separated by ~150 kb and we predict that nine class III genes found in the opossum fall into this gap in the wallaby MHC. Similarly, Contigs 5 and 6, containing framework genes, are separated by ~250 kb based on comparison with the homologous opossum region.

Contigs 7, 8 and 9 contain a cluster of class II genes belonging to the DAB gene family interspersed with DAB pseudogene fragments. These contigs map (by FISH) to a region ~1 Mb telomeric of the framework region (Figure 2), but we could not determine the exact order of the contigs. The contigs contain 11 unique class II DAB sequences. However, as the DAB genes and surrounding pseudogenes share high sequence similarity it is possible the BACs are misassembled and are actually hybrids of the two haplotypes present in the BAC library. The two orphan BACs containing class II DAB, DBB and DAA genes map (by FISH) to this region and do not overlap with Contigs 7, 8 or 9.

### Wallaby TAP Genes

**Figure 4:**
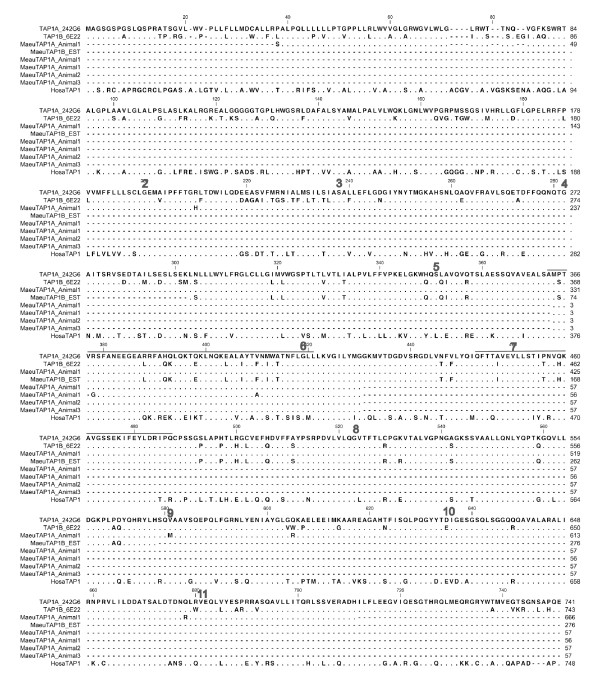
**Alignment of TAP1 genes from the wallaby and expressed transcripts isolated from spleen, blood and EST library**. Amino acid alignment of TAP1 sequences. TAP1A_242G6 and TAP1B_6E22 are sourced from BACs in Contigs 1 and 2 respectively. MaeuTAP1A_Animal1 was sourced from a the spleen of animal1, TAP1B_EST is sourced from a mixed tissue EST library. MaeuTAP1A_Animal1, MaeuTAP1A_Animal2 and MaeuTAP1A_Animal3 were sourced from blood samples from Animals 1, 2 and 3 respectively. HosaTAP1 is included for comparison. Dashes indicate missing sequence, while dots indicate conserved residues. Exon boundaries are indicated by a number above the first residue of the exon. Residues thought to interact with the peptide are indicated by a line above the sequence.

**Figure 5:**
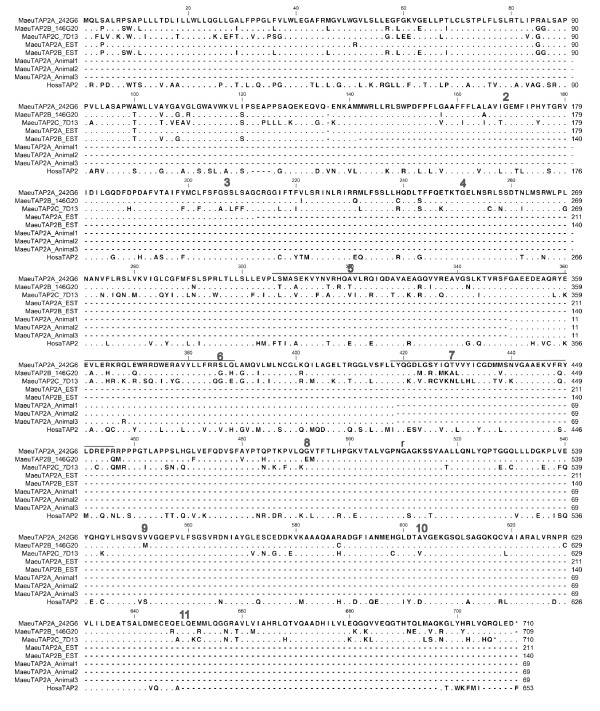
**Alignment of TAP2 genes from the wallaby and expressed transcripts isolated from spleen, blood and EST library**. Amino acid alignment of TAP2 sequences. TAP2A_242G6, TAP2B_146G20 and TAP2C_6E22 were sourced from BACs in Contigs 1 and 2. MaeuTAP2A_EST and MaeuTAP2B_EST were sourced from a mixed tissue EST library. MaeuTAP2A from Animals 1, 2 and 3 were sourced from blood samples. HosaTAP2 is included for comparison. Dashes indicate missing sequence, while dots indicate conserved residues. Exon boundaries are indicated by a number above the first residue of the exon. Residues thought to interact with the peptide are indicated by a line above the sequence.

**Figure 6:**
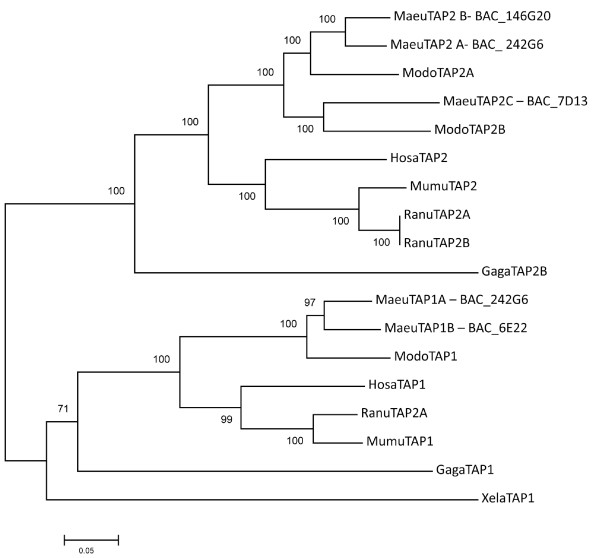
**Neighbour-joining phylogenetic tree showing the relationship between TAP1 and TAP2 genes from the wallaby and other vertebrates**. Analysis was performed on the full amino acid sequence for each gene.

Within the wallaby MHC class I region there has been an expansion of the antigen processing genes, TAP1, TAP2 and PSMB. The TAP1 genes with complete open reading frames are found on BAC 242G6 (TAP1A-Contig 1) and BAC 6E22 (TAP1B-Contig 2), while the complete TAP2 genes are found on BAC 242G6 (TAP2A), BAC 7D13 (TAP2C) and BAC 146G20 (TAP2B) (Figure 4 and 5). The TAP genes are interspersed with non-classical class I genes and the DMA and DMB genes. Phylogenetic analysis shows that the TAP2 genes on BAC 242G6 (Contig 1) and 146G20 (Contig 2) cluster together and share 89% amino acid identity (Figure 5 and 6). TAP2C, located on BAC 7D13 is more divergent, but is more similar to the opossum TAP2B gene. It shares 76% amino acid identity with the wallaby TAP2 genes and 79% amino acid identity with the opossum TAP2B. The TAP1A and TAP1B genes on BAC 242G6 and 6E22 cluster together and share 87% amino acid identity with each other and 81% and 77% identity respectively with opossum TAP1.

The TAP promoters were examined by aligning the 200 base pairs of sequence upstream from the putative transcriptional start sites with the promoter sequences from human and opossum TAP genes. The TAP1A and TAP1B genes share 70% nucleotide identity across this region, emphasizing the distinctiveness of these genes from one another. The TAP1A and TAP1B genes share 47 and 50% identity respectively with the same regions of the human TAP1 gene. A potential ISRE, NF-kB site and GC sites were identified for both TAP1B and TAP1A. The TAP2A and TAP2B genes share 80% nucleotide identity across the promoter region and 50-55% identity with the homologous region of TAP2C (data not shown).

To determine whether all of the TAP1 and TAP2 genes are expressed, primers were designed to amplify exons 5 and 6 as the sequences of the two paralogs differ in this region (summarized in Table 2 and Figures 4 and 5). Transcripts that share between 97-100% nucleotide identity to TAP1A (BAC 242G6) were detected in the spleen from one animal and the blood from two additional animals. Only transcripts sharing 99% nucleotide identity to TAP1B were isolated from a mixed tissue EST library constructed from a fourth animal, but no TAP1A transcripts were isolated from this library. This means that expression of TAP1A and TAP1B were not detected in the same animal or in the same tissue type (Table 2). In contrast, TAP2A (BAC 242G6) transcripts were found in spleen and blood samples and both TAP2A and TAP2B were isolated from the EST library. Transcripts for the TAP2C gene on BAC 7D13 were not identified in any tissues or the EST library.

**Table 2:** TAP variants found in spleen, blood or combined tissues from four different animals.

TAP1	Animal 1 Spleen	Animal 2 Blood	Animal 3 Blood	Animal 4 Combined tissue ESTs
TAP1A_242G6	Yes	Yes	Yes	No

TAP1B_6E22	No	No	No	Yes

**TAP2**				

TAP2A_242G6	Yes	Yes	Yes	Yes

TAP2B_6E22	No	No	No	Yes

TAP2C_7D13	No	No	No	No

### Rearrangement of the MHC region

A KERV (Kangaroo Endogenous Retrovirus) fragment was identified next to the non-MHC pseudogenes at position 569 kb of Contig 2 on BAC93J23. KERV fragments were also identified adjacent to the class III region and the VAMP4 pseudogene on BAC198J4 and BAC163H18 and next to the class II DAB cluster on BAC 178C11 and the DBB genes on BAC 171E14.

### The class II antigen presenting genes have duplicated and form two clusters separated by the class III genes

MHC class II genes are found on nine of the sequenced BACs (Figure 1) and include at least one DMA, one DMB, seven DAB, four DBB, one DAA and two DBA genes. A pseudogene similar in sequence to the opossum DCB gene was identified with in-frame stop codons in the β1 and β2 domains.

The classical class II genes are found in two regions: the first lies between the antigen processing genes and the class III genes, and contains the DBA, DAA and DBB genes. The second is found at the telomeric end of the region and contains DAB genes as well as additional DBB and DBA genes.

**Figure 7:**
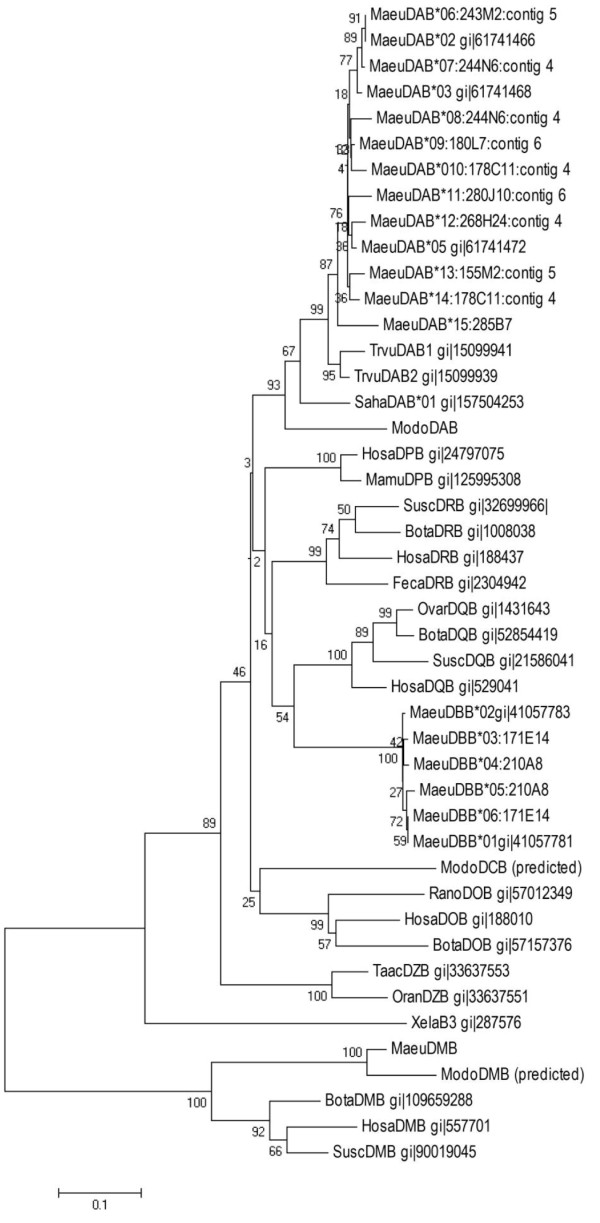
**Neighbour joining phylogenetic tree showing the relationship between mammalian class II B chain genes**. Analysis was performed on the amino acid sequence from the β2 domain only.

There is a minimum of 7 and a maximum of 10 DAB loci, however, heterozygosity in the individual from which the BAC library was made and the complexity and polymorphism of this gene family means the number of DAB loci is difficult to resolve. Five DAB genes are found on Contig 7. Four are found on Contigs 8 and 9. A further DAB gene is located on BAC 285B7. All of these DAB genes have complete open reading frames and contain residues consistent with functional genes. As many of the DAB sequences are closely related (share between 83 and 92% nucleotide identity across the entire coding region of the gene) and are interspersed with pseudogenes and DAB gene fragments it is difficult to determine if some identified DAB genes are representative of different haplotypes. For example, we predict that Contigs 7 and 8 could represent different haplotypes as 244N6.5 (Contig 7), 243M2.1 (Contig 8), 178C11.2 (Contig 7) and 155M2.3 (Contig 8) are highly similar DAB genes. Contig 9 is more difficult to place and may represent additional loci or alleles. This suggests that wallabies have 6-8 DAB loci. Comparison with known wallaby DAB transcripts indicates that at least five of these genes are expressed (Figure 7). The expression of the remaining genes is unknown. Interestingly, a DAB processed pseudogene was found on Contig 3 between the antigen processing genes and the class III genes. The pseudogene lacks introns, but cluster with the other DAB genes in a phylogenetic tree (data not shown).

The DBB, DBA and DAA genes have been physically mapped to two different locations separated by the class III region. BAC_171E14 containing two DBB genes and one DBA gene lies adjacent to the DAB gene cluster telomeric to the MHC. A second BAC (210A8), containing an additional DBB gene, a DBA gene and a DAA gene lies between the class I/antigen processing genes (Contig 2) and the class III region. No intact class II genes are found on Contig 2. The corresponding region to Contig 2 in the opossum contains DBB, DBA and DAA genes located adjacent to the class I genes, *Modo-UM, Modo-UE *and *Modo-UL *[18]. However, Contig 2 contains a single class II α chain gene fragment with two adjacent β chain gene fragments (Figure 3), suggesting that the genomic organization in ancestral marsupials was more akin to that seen in present day opossums.

We predict the wallaby has four DBB genes, one DAA gene and two DBA genes. The wallaby DBB genes share high levels of sequence similarity, between 92 and 94% nucleotide identity across the entire coding region, and 83 and 88% nucleotide identity across the peptide binding region. Phylogentic analysis shows that the DBB genes form a species specific clade and *MaeuDBB1 *and *MaeuDBB3 *are closely related to previously isolated DBB cDNA clones (Figure 7) [26].

## Discussion

The wallaby MHC has undergone extensive rearrangement since the divergence of the Australian and American marsupials. The classical class I genes have moved out of the core MHC region on chromosome 2q and are found at 10 separate chromosomal locations [24]. Remnants of a class I/II region are visible on 2q, but this region now only contains non-classical class I genes and duplicated antigen processing genes. The class II MHC genes have relocated into two clusters, which are separated by the class III region and the extended class I region. This unique MHC organization allows us to pose questions about the importance of gene clustering for antigen processing and presentation and explore how the mammalian MHC evolved.

### The classical class I and antigen processing genes are not linked in the wallaby

Linkage of classical class I and antigen processing genes within the MHC has been shown to have functional significance in vertebrates. This is most pronounced in non-mammals [13,15] and the rat [10] where classical class I and TAP genes are adjacent to one another, resulting in minimal recombination. Some species with this type of MHC gene organization, such as the chicken, have only a single class I gene allowing co-evolution of class I and TAP alleles that can work together to process and present specific peptides [17]. In chickens, the TAP genes are polymorphic and in the rat there are multiple lineages of TAP2, resulting in a necessary co-evolution between class I and TAP alleles that has direct functional consequences on the peptides that are processed and presented to T cells by each MHC haplotype [10,17,27]. In contrast, most eutherian mammals have multiple classical class I genes that are separated from the antigen processing genes by the class III region. It has been proposed that loss of tight linkage between class I and antigen processing genes may have facilitated the expansion of the classical class I in mammals [17,28].

We have previously shown that classical class I genes are not found within the core MHC on wallaby chromosome 2q [24]. We found only non-classical class I genes in close proximity to antigen processing genes. A single class I pseudogene, which is most similar to the classical class I genes outside the MHC (rather than the non-classical class I on chromosome 2q), was identified within this region. This implies that the classical class I genes were once found within the MHC, but subsequently moved away. The movement of classical class I genes away from the antigen processing genes most likely had implications for how these genes evolved and may have facilitated the expansion of both the classical class I and TAP gene families in the wallaby.

Most vertebrate species have only a single TAP1 and TAP2 gene, which form a heterodimer on the ER membrane. In mammals the TAP molecules are generally permissive in the peptides they pump into the ER and it is the class I molecules that are selective in the peptide they will bind. The wallaby has multiple TAP1 and TAP2 genes. It appears that TAP2B (BAC 146G20) and TAP1B (BAC 6E22) arose via duplication events from the TAP1A and TAP2A genes on 242G6 (Figure 6). The TAP2C gene on BAC 7D13 is orthologous to opossum TAP2B and may have been present in the marsupial ancestor. However, we found no evidence that TAP2C is transcribed. We found evidence that both TAP1A and TAP1B are transcribed, but we did not find these genes expressed in the same animal or the same tissue type. This may mean that these genes are differentially expressed in different individuals or different tissue types. In contrast, there is evidence that both TAP2A and TAP2B transcripts are expressed in a single individual, but in different tissue types. As a whole this data indicates that diversity is generated among functional wallaby TAP molecules. How the TAP genes are coordinated in the wallaby cannot yet be determined. We have considered two possibilities. First, the TAP1 and TAP2 genes may co-ordinate in a random manner. This is supported by the finding of multiple TAP2 transcripts in the same individual. This type of interaction may allow a wider range of peptides to be pumped into the ER and in turn be presented by class I. Second, the TAP genes may interact in a specific manner, and may pump peptides for binding to specific class I genes or only in certain tissues, increasing the specificity of peptides pumped into the ER. This hypothesis seems more likely as the TAP2A and TAP2B genes are expressed in distinct tissues in the same individual. The system utilized by the wallaby may represent a new way for TAP genes to provide specificity or promiscuity in the peptides provided to class I molecules.

### Genomic organization of class II

In some non-mammals, class II genes are located in a single cluster next to the class I genes. Similarly, the opossum, which last shared a common ancestor with the wallaby ~80 mya contains a class I/II region [18] and in the platypus, which last shared a common ancestor with the marsupials and placental mammals ~160 mya [29], the classical class II genes are adjacent to class I and antigen processing genes [30]. In the wallaby the class II genes have undergone rearrangement and the classical class II genes are divided into two regions. The first class II region, containing DBB, DBA, DAA genes and DAB pseudogenes, is adjacent to the antigen processing genes. We propose that this was the class II region in the common marsupial ancestor. A second class II region is located towards the telomeric end of the chromosome. This cluster contains the DAB genes, two DBB genes and a DBA gene.

### Class II copy number

The class II genes have undergone large scale expansion and rearrangement since the divergence of the American and Australian marsupials. The tammar wallaby is the only marsupial species for which the number of DAB genes has been determined using large scale sequencing, as the DAB genes were not sequenced in the opossum genome, and only a single gene has been characterized at the cDNA level [31]. Nevertheless, it is still difficult to determine the exact number of genes present in the wallaby genome due to the presence of two haplotypes in the individual from which the BAC library was made and the close sequence identity between the DAB genes. We predict that the wallaby has at least seven DAB genes. Four DAB genes have been identified in *Gracilinanus microtarsus *and two DAB genes were identified in *Marmosops incanus*, two species of Brazilian mouse opossum [32]. Among the Australian marsupial species the brushtail possum (*Trichosurus vulpecula*) [33] has at least five DAB genes, while the Tasmanian devil (*Sarcophilus harrisii*) has at least three DAB genes [34].

Only a single DAA gene was identified 1 Mb away from the cluster of DAB genes and two DBA genes were identified. Similarly, there is evidence for a single DAA gene and multiple DBA genes in the brushtail possum [33]. The wallaby has at least three DBB genes [35], whereas there are only two in the opossum. It has been predicted that the brushtail possum has at least two DBB genes and DBB transcripts have also been isolated from the red-necked wallaby [33,36].

### Class II heterodimers

Based on the organization of the class II genes in the wallaby we predict that the DAB and DAA genes form heterodimers, while the DBB and DBA genes most likely form heterodimers. Holland and colleagues (2008) recently proposed that in brushtail possums, highly variable DAB genes form heterodimers with the almost monomorphic DAA genes and the somewhat polymorphic DBB genes form heterodimers with the DBA genes [33]. This is reminiscent of the relationship between the DR α and β gene pairs in eutherian mammals, where one member of the partnership is highly polymorphic, while the other is not. It has been proposed that the genetic distance between α and β chain genes and the amount of recombination defines the level of polymorphism of a class II gene [37]. Where there is a sufficient amount of recombination between α and β genes one member of the partnership (usually the α gene) must remain monomorphic so that it can form a complex with any number of β gene alleles. Conversely, where there is little recombination between the genes, alleles may co-evolve and there is no reason for the α gene to remain monomorphic. For instance, it has been proposed that frequent recombination within the mouse class II region between H2-Eα and H2-Eβ genes, results in a highly polymorphic H2-Eβ and nearly monomorphic H2-Eα, which can form a complex with any of the β chain genes [37]. Similarly, in chickens a single monomorphic class II α gene is separated by at least 50 kb from the polymorphic β gene, with the proposal that this genetic separation has allowed the β chain to be highly polymorphic and forced the α chain to become monomorphic and a best fit to the β chain [38]. In the wallaby the single DAA gene is separated from the DAB family of genes by at least 1 Mb and the gene dense class III region. Here we present evidence that the DAB gene family has multiple expressed genes. We speculate that the DAA locus in the wallaby will be non-polymorphic, so that it can form functional dimers with the highly variable DAB family. In contrast, there are tightly linked DBA and DBB genes in both of the wallaby class II regions, suggesting that these genes can more easily co-evolve to generate functional dimers. This is supported by evidence of polymorphism at DBB genes in both the wallaby [25] and in DBB and DBA genes in the brushtail possum [33]. However, further data on polymorphism in wallaby class II genes is needed. The movement of DAB genes away from the DAA gene may have allowed the DAB gene family to expand rapidly and is perhaps reminiscent of the class I genes in the wallaby, which we predict moved away from the antigen processing genes and then expanded to create multiple classical class I.

### Kangaroo endogenous retrovirus and gene rearrangements

Kangaroo endogenous retrovirus (KERV) was originally discovered due to its role in macropod chromosome rearrangement and evolution [39]. We previously identified KERV fragments adjacent to class I genes that have moved away from the core MHC and speculated that these elements played a role in the movement of class I genes, as has previously been identified in eutherian mammals [23,24,40]. Here we identified KERV fragments within the rearranged class I/II region and adjacent to *NOTCH4 *in the class III region, implying that retroviruses have played a key role in the evolution of the wallaby MHC. We have also identified a class II DAB pseudogene that lacks introns, but is otherwise intact and with a putative PolyA tail, adjacent to the rearranged class I/II region. Retroviral activity may have played a role in the evolution of the wallaby MHC, by moving DAB genes away from their DAA counterpart, resulting in the expansion of the DAB gene family and leaving traces of their activity in intronless class II pseudogenes.

In a broader context, analysis of retroposon insertions within the South American and Australian marsupial orders has shown that the Australian marsupials derived from a single common ancestor, indicating a single marsupial migration from South America to Australia [41]. Most interestingly, the analysis also implies a high degree of retroposon activity in the lineage leading to the modern Australian marsupial orders. It is possible that the derived nature of the wallaby MHC (in comparison to the opossum) is in part due to the activity of these retroposons in the common ancestor of the Australian marsupials. From this interpretation it follows that the divergent organization of the wallaby MHC, including the unique organization of the class I and class II genes, may be common to the Australian marsupial species.

## Conclusions

The wallaby MHC has undergone extensive rearrangement since this species shared a common ancestor with the South American marsupials. Although the remnants of a class I/II region, seen in the opossum and non-mammals, are visible in the wallaby there are no classical class I genes within the MHC. This is remarkable for a mammalian MHC and may have affected the number of antigen processing genes and their expression, resulting in multiple combinations of TAP heterodimers. The movement of class I and class II genes may have facilitated the generation of diversity within these gene families. The wallaby class II genes are found in two regions separated by the class III genes and this most likely triggered the expansion of the highly polymorphic class II DAB family of genes. Analysis of the wallaby MHC has provided insights into the evolution of this gene family in marsupials and shed light on factors that have influenced the evolution of the MHC in this branch of mammals.

## Methods

### Selection of MHC-associated BAC clones

Forty-nine overgo probes were designed based on annotated opossum MHC genes (Additional File 1, Table S1), which were extracted from the opossum MHC genome browser (available at: http://bioinf.wehi.edu.au/cgi-bin/gbrowse/opossum_mhc/). These MHC genes were used to search against the tammar wallaby genome trace archive (2 × coverage deposited on the NCBI database) using a discontinuous BLAST (Basic Local Alignment Search Tool) for cross species searches. Significant matches from the wallaby trace archive were searched against the Genbank database to confirm the identity of the sequence. Overgos were designed for each tammar trace sequence using OvergoMaker [42]. All overgos were 24 base pairs in length, with an overlap of eight base pairs and a GC content of 45-55%. After a preliminary contig was built (see below for methods) ten BACs were selected for BAC end sequencing (T7 and Sp6 primers). Thirty additional overgos were designed from this sequence (Additional File 1, Table S1).

Overgo probes were radio labeled with ^32^P-dATP and ^32^P-dCTP (GE Healthcare) and used to screen a 11 × tammar wallaby BAC library (Me_KBa, Arizona Genomics Institute, USA) using the BACPAC hybridization protocol (available at: http://bacpac.chori.org/overgohyb.htm). The following modifications to the protocol were made; overgo probes were pooled in groups of ten and used to screen six filters at once. Following hybridization and washing, the filters were exposed to Hyperfilm (GE Healthcare) using intensifying screens for up to fourteen days at -80°C.

### Secondary Screening of MHC associated BAC clones

Secondary screening of positive BAC clones was used to determine BACs positive for individual MHC genes and to remove false positive clones. BAC clones were cultured overnight and 1 ul of culture was applied to gridded Hybond N+ membrane (GE Healthcare). The membranes were placed on LB/agar plates with chloramphenicol (12 μg/ml) and incubated at 37°C overnight. The membranes were removed from the plate and placed on blotting paper moistened with a denaturing solution for 7 min, followed by blotting paper moistened with a neutralizing solution for 7 min. The membranes were rinsed in 2 × SSC and baked for 2 hours at 80°C. Ten overgo probes (Additional File 1, Table S1) were radiolabeled as described above and used to screen these membranes at 60°C overnight. Membranes were washed according to the BACPAC hybridization protocol and membranes were exposed to Hyperfilm (GE Healthcare) using intensifying screens overnight at -80°C.

### Physical Mapping of BACs

Many MHC class I genes of the tammar wallaby are known to be located outside the MHC. Thus, BACs known to contain class II MHC genes were physically mapped using Fluorescent In Situ Hybridization (FISH) according to Deakin et al. (2007) [23]. Dual-colour fluorescence in situ hybridisation (FISH) was used to determine the location of orphaned BACs or BAC contigs. BACs were labeled by nick translation with either SpectrumOrange or SpectrumGreen (Vysis), hybridised to male tammar wallaby metaphase chromosome spreads and imaged as described in Deakin et al (2008).

### Interphase nuclei preparations

A male tammar wallaby fibroblast culture was grown to confluency and held without medium change for 3 days to enrich for G_1 _interphase cells. Cells were harvested by trypsinisation, washed twice in PBS, swollen in 75 mM KCl at 37°C for 15 min, fixed in 3:1 methanol: acetic acid and dropped onto glass slides. Three separate experiments were performed on interphase nuclei for each orphaned BAC. BAC 310P15 representing the framework region/Class III contig and 288B16 representing the extended region were directly labelled by nick translation with SpectrumGreen (Vysis) and the orphaned BAC was labelled with SpectrumOrange (Vysis). In experiment one all three BACs hybridised to nuclei in the same experiment elucidated whether orphaned BACs were within the MHC spanning from the Class III region to the extended Class I/II region. Two further experiments were carried out with either BAC 310P15 or 288B16 labelled with SpectrumGreen and the orphaned BAC labelled with SpectrumOrange allowed the orientation of the orphaned BAC in relation to the flanking region BACs to be ascertained. Hybridisation of labelled probes to interphase nuclei was carried out following the FISH hybridisation protocol described in Deakin et al (2008). A total of 50 nuclei were imaged for each interphase experiment.

### BAC contig assembly

All BAC clones were assembled into contigs using BAC fingerprinting as described by Marra *et al *(1997) [43] and Humphrays *et al*. (2001) [44] followed by contig analysis using FPC (v6.5) [45]. Known MHC markers on the BACs identified by secondary screening were used to assess the validity of any contig merges. BACs constituting a minimum tiling path were then selected for sequencing. We used the opossum MHC as a guide to ordering the contigs, but with some caution, as we have previously shown the organisation of the wallaby class I genes is very different to that of the opossum [24].

### Sequencing of overlapping BACs

Sequencing of BACs occurred at the Wellcome Trust Sanger Institute as previously described [46]. The BACs for which sequencing and annotation have been completed have been submitted to Genbank under the following accession numbers. MEKBa_288B16 [CU463226]; MEKBa_466E14 [CU463226]; MEKBa_47C8 [CU464026]; MEKBa_293I1 [FP104545]; MEKBa_242G6 [CU463018]; MEKBa_49O16 [CU463996]; MEKBa_93J23 [CU463939]; MEKBa_7D13 [CU464027]; MEKBa_6E22 [CU463963]; MEKBa_146G20 [CU466525]; MEKBa_241L16 [CU463962]; MEKBA_212C16 [CU463025]; MEKBA_189L19 [CU463023]; MEKBa_210A8 [CU464025]; MEKBA_163H18 [FP104544]; MEKBA_460F7 [FP236778]; MEKBA_198J4 [FP236847]; MEKBA_458G11 [FP236731]; MEKBA_575K20 [FP236744]; MEKBA_5M36 [FP236732]; MEKBA_310P15 [FP236629]; MEKBA_455E20 [FP236651]; MEKBA_231N5 [FP236650]; MEKBa_180L7 [CU468126]; MEKBA_280J10 [CU467811]; MEKBA_244N6 [CU464032]; MEKBa_268H24 [CU463175]; MEKBA_178C11 [FP016133]; MEKBa_171E14 [CU464024]; MEKBa_285B7 [CU463152]; MEKBa_243M2 [CU463026]; MEKBa_155M2 [CU463961]. A previously sequenced BAC (VIA_6605) containing class III genes was also included in the contig [47]. The full annotation and sequence for each BAC can be found at http://vega.sanger.ac.uk/Macropus_eugenii/Info/Index.

### Phylogenetic and sequence analysis

The overlapping regions of fully sequenced BACs were determined using Sequencher 4.1.4 (GeneCodes) with 10% minimum overlap and 80% minimum nucleotide identity. The overlapping regions were then checked manually for mismatches. The predicted, full length coding sequences of the MHC class II α chains and β chain and TAP genes were aligned with the sequences from the NCBI database listed below using ClustalW, in the Bioedit program [48]. Neighbour joining trees were constructed with the β2 domain of the class II genes and the full amino acid coding sequence of the TAP genes using the Jones-Taylor-Thornton matrix and 1000 bootstraps in the Mega 4.0 software [49].

TAP sequences used for phylogenetic analysis were as follows: Opossum: ModoTAP2A, ModoTAP2B and ModoTAP1 can be found at http://bioinf.wehi.edu.au/opossum/seq/Class_II.fa; Human: HosaTAP2-[M74447], HosaTAP1-[X57522]; Mouse: MumuTAP2-[M90459], MumuTAP1-[U60018]; Rat: RanuTAP2A-[X638854]. RanuTAP2B-[CAA53055], RanuTAP1-[X57523]; Chicken: GagaTAP2B-[AJ843262], GagaTAP1-[AJ843261]; Xenopus: XelaTAP1-[AF062387].

MHC class II β chains sequences used for phylogenetic analysis were as follows: Brushtail possum: TrvuDAB, AF312030; Red-necked wallaby: MaruDAB*1-[M81624]; MaruDBB-[M81625]; Tammar wallaby: MaeuDAB*5- [AY856414]; MaeuDAB*2-[AY856411]; MaeuDAB*3-[AY856412]; MaeuDBB*1- [AY438038]; MaeuDBB*2- [AY438039]; Tasmanian devil: SahaDAB*01-[EF591102]; Opossum: ModoDAB -[AF010497]; ModoDBB1, DCB and DMB can be found at http://bioinf.wehi.edu.au/opossum/seq/Class_II.fa; Platypus: OranDZN-[AY288074]; Echidna: TaacDZB1-[AY288075]; Human: HosaDOB-[M26040]; HosaDPB1-[NM002121]; HosaDMB-[AK295872]; Cow: BotaDRB-[D45357]; Pig: SuscDRB-[AY191776]; SuscDQB-[AY102478]; SuscDMB- [NM_001113707]; Horse: EqcaDQB-[L33910]; Cat: FecaDRB-[U51575]; Sheep: OvarDQB- [L08792]; Chimpanzee: PatroDOB- [M24358]; Gorilla: GogoDRB-[M77152].

### Analysis of TAP1 and TAP2 expression

Primers were designed to amplify exons 5 and 6 of the wallaby TAP1A, TAP1B, TAP2A, TAP2B and TAP2C genes (TAP1F-CTGTGGAGGCACTTTCTGC, TAP1R-. CATCGGTCACCATCTTTCC, TAP2F-TTGGAGCAGAGGAGGATGA, TAP2R-GAGTAGGAATGAGACAAGGC). The primers were designed to regions where the multiple TAP1 and TAP2 genes are identical, but across a region that would allow different genes to be identified. TAP1 and TAP2 fragments were amplified from a spleen sample and blood samples (n = 3) with the following reaction: 1 × Buffer, 2 mm MgCl_2_, 200 μm dNTP, 2 μm of each primer and 0.3ul of High fidelity taq polymerase (Expand taq, Roche). Cycling conditions were as follows: Initial denaturation at 94.0°C for 3 min, followed by 29 cycles of 94.0°C for 30 s, 57°C for 30 s, and 72°C for 40 s, and a final extension at 72°C for 10 min. A 250 base pair fragment was amplified and cloned into a commercial vector (Clonejet, Fermentas). Twelve clones were selected from each spleen or blood sample and sequenced using a M13F and M13R primers. A 5' and 3' EST database constructed from mixed tissue of a single wallaby (including spleen and lymph node) was blasted using full length TAP1A, TAP1B, TAP2A, 2B and 2C sequences. Access to the library was kindly provided by Marilyn Renfree at the ARC Centre of Excellence in Kangaroo Genomics.

## Authors' contributions

KB and SB designed the project. HVS isolated BACs, carried out phylogenetic and sequence analysis of MHC genes and drafted the manuscript. JED isolated BACs and carried out FISH experiments. PC fingerprinted and processed BACs. LW and JH annotated BACs. JK contributed to analysis of TAP genes and generation of TAP transcripts. All authors edited and approved final manuscript, with particular help with figures from LW.

## Supplementary Material

Additional file 1 **Table S1**. Overgo probes used for BAC isolation. Click here for file
