# Supplementary material for: The tammar wallaby major histocompatibility complex shows evidence of past genomic instability
Source: BMC Genomics. 2011 Aug 19;12:421. doi: 10.1186/1471-2164-12-421 (PMC3179965; doi:10.1186/1471-2164-12-421)
Supplement: Additional file 1 — Table S1. Overgo probes used for BAC isolation. [file 1471-2164-12-421-S1.PDF]

Supplementary Table 1. Overgo probes used for library screening.

Gene	Overgo	Gene	Overgo
LSM	F- CTCAACATCAAACTAACAGATATC<br>R-CTGGGTCTGTAACACTGATATCTG	NRM	F- TCTCCCCCCATCATAAGATTCTCG<br>R- AGTCAGCCATTCCTGTCGAGAATC
RING1	F- CTCTCCAAGTACTTGGCTCTAAGG<br>R- TCCGCTCAAGTGCAATCCTTAGAG	TAPBP	F- TGGTCTAACCCCTGAACCTTACTC<br>R- ATCCAATGACCGAGGAGAGTAAGG
APOM	F- TGTGTACCCCGAGAATGGACTTAC<br>R- TCCCTTCAGTCAGATGGTAAGTCC	DOM3Z	F- GAACTTCTGTGCTGCTTTCCTCAG<br>R- TGTGCTTTGGGCAAAGCTGAGGAA
RGL2	F- TTCCAGACTAGAGACCCTAGAGTC<br>R-ATCATCACAAGGCCTGGACTCTAG	NOTCH4	F- TGCTAGTTGCCCATCCCCAAGAAG<br>R- TAGCATCTACCTGTGCCTTCTTGG
PBX	F- ACTCCATTGAGCACTCCGACTATC<br>R- TGGGCCAGTTTACTTCGATAGTCG	BF	F- ATTTGGAATTGGGCCCCTGGTAGA<br>R-GTTGATCTTCTCCTCGTCTACCAG
CREBL1	F- ACTTCCAGACCTACAGGTGAAGTC<br>R-TGGGGAAGAAGGTTCTGACTTCAC	RDBP	F- GGACCAAGGTAGAATCTGTGCAAC<br>R- GCGATGCTAACCTTGAGTTGCACA
PHF	F- TGCTGGGGATGAAGCTGTCTTTGC<br>R- CCCTTCAGTCCATATGGCAAAGAC	PSMB9	F- GCAGTGGAGTTTGATGGTGGTGTC<br>R- AATCAGAACCCACCACGACACCAC
SKIV2L	F- CTCGACCTTACTACTAGTTCTGAG<br>R- GGTGGATCTCACTCTTCTCAGAAC	BRD2	F- GTCTCACACACCTTTGTATTCCCC<br>R- GGGAATCTCAGGTGATGGGGAATA
TAP1	F- AAAGGGCTGACCACATACTCTTCC<br>R- ATGACCCCCTCCTCTAGGAAGAGT	SYNGAP1	F- CTGCACAAGTAGCTCAGACATCAC<br>R- CTTCTGTTCTGGCTCAGTGATGTC
TAP2B	F- ATGGGAAGCCCTTGGTTGAGTACC<br>R- TGCAGGTATTGGTGCTGGTACTCA	CLIC1	F- TTCTATCCCAGAGGAGTTTCGAGG<br>R- CAGGTATCTTTGCACCCCTCGAAA
VPS52	F- CCTCATGACTTCTCTCTGGGTCTA<br>R- TTGCCTGAATGTGAACTAGACCCA	NEU1	F- TTCCACCATGCTGGTGTGGAGCAA<br>R- AGTGATGCCATCATCCTTGCTCCA
POU5F1	F-GCAAACGCAGCAATAGCAACTCAC<br>R- TCGAAATCTTCTCGGGGTGAGTTG	MRPS18B	F- AGTTGGAGGCAACAACTCGATACC<br>R- GTCTCTTCTGGCATGTGGTATCGA
CDSN	F- CCTCCTCCAATTCCTATGGTAGCT<br>R- CCACCTACCACTTCATAGCTACCA	DHX16	F- GCTGTTCAGAGGAGATCCTTACAG<br>R- GATAACATGGCAGCCACTGTAAGG
TCF19	F-TGGGAAGGGTGGAGATCTCTATAC<br>R-TGGGAGGGTGAAAAGTATAGAG	ABCF1	F- AGAACCAGGATGAGGAGGCACAAG<br>R- AAAAGCTCAGGTGCCTCTTGTGCC
KIFC	F- CTGGATGATTACCCTGTGTGCATC<br>R-TCTGGCCATAGGCAAAGATGCACA	GNL1	F- ACCCACTGTTCGGCTTTGTGACTG<br>R- AAAGATAAGGCCAGGGCAGTCACA
RXRB	F- TATCTCCTCTGATCCCCATCTCCT<br>RGGCAAAAGAGAGGAAGAGGAGAT	RPS18	F- AGTGCTCAACACCAACATTGACGG<br>R- AGCTATTTTCCGCCGTCCGTCAAT
Vars2L	F- CATCATCTTGCCCAGTGTTGTTGC<br>R- TTGGGCTCTGAACATTGCAACAAC	B3GALT4	F- CTTATGGTTCAGGCACTGGCTATG<br>R- ACTGGGGCTGATAACACATAGCCA
FLOT1	F-TAAGCCACCTGCCAAATTGTGTGG<br>R-ACACCTGTGAGCTTCTCCACACAA	ZNF297	F- AGCTGAGTGATGATGGAGGGGATG<br>R- ACTACTACCCCAACTCCATCCCCT
ZBTB12	F-GCCTAAAAGAGGATGGAGTCAGTG<br>R-GTCAATCCAATCCCTCCACTGACT	DMA	F- CTGATGTCTTTGGTCCATCTTCCC<br>R- TGATGCCAACACTGCTGGGAAGAT
BAT8	F- AAAGCGGGAAACGATGAGGCTTTC<br>R- CAGAGCTACTCTGCTAGAAAGCCT	DMB	F- CCCACAGGACACGTAAGAAGGAAG<br>R- TTTCTTTCTGCAGCCCCTTCCTTC
SLC44A	F- CAGGTCTAACTTGCCAGTTCCTAG<br>R-CCTGAAGAGGAGTAACCTAGGAAC	DXA	F- AGTGAGTTACCTCACCTTCATCCC<br>R- GACACCTTCAGTGGAAGGGATGAA
MOG	F- GAGTGGTTCACCTCTACCGAAATG<br>R-CCATCTTGATCCTTCCCATTTCGG	DCA	F- GAGTTTGGCCGTATCTTCAGTTCC<br>R- GCCCAATCTGTGCATTGGAACTGA
GABBR1	F- TGCTGTCACAGATGCTTAGCTTCA<br>R- CATTCCCCTAAAGAGATGAAGCTA	DCB	F- GAGGAGATGTCTTTGCTTGCCATG<br>R- AAGCTGACATGGTCCACATGGCAA
RPP21	F- CTGGCGAGGTTTTACTGCCACACG<br>R- TGCCGATGGTTCTTTCCGTGTGGC	SLC39A7	F- GCACTCCCATGAAGATTTCCAGCA<br>R- GCACTCCCATGAAGATTTCCAGCA
C4B	F- TCTGAGGATGTGTCCTTTACCCTG<br>R- AGTCATGGTCAGAGTCCAGGGTAA	155P10sp6	F-TGCCCCTGACACATACTGGCTTTG<br>R- GACTTACCCAGGGTAACAAAGCCA
146G20sp6	F- CTTGGCATATGGTGTAAGATGGTG<br>R- GCAATGAGGCATAGACCACCATCT	178C11sp6	F- AATTGATGCCATCTGAAGTTATTG<br>R- GCCCAATTCACAACAGCAATAACT

201022sp6	F- TGTTAGTGAGGCAGTGAGGATGCC<br>R- CTATACCAACCACCTGGGCATCCT	242G6sp6	F- GTGTGGCCATAAACTCTGGTGTTC<br>R- GCAAGGAAGAGCACTAGAACACCA
243m2sp6	F- AGTTCCAGACTTGCCTGTGGATTC<br>R- CAACACGTAGGACTTCGAATCCAC	244N6sp6	F- TCAAACACCTAGGGACGTACTGAG<br>R- GGAAGCAGCAGTGTAACTCAGTAC
180L7t7	F- CAGCAAAGACTGATGGCATTCATC<br>R- CCCTCTCAGTAAGTAGGATGAATG	242G6t7	F- ACAGAAAGCAGGCAGAACTGAAGG<br>R- CTGCTTCCTGCTTTCTCCTTCAGT
243m2t7	F- CCAGGGCTATGCGAATGAGGTTTC<br>R- CAGGATTCCTCTACAGGAAACCTC		
